# Supplementary figures and images for: Production of fatty acids in Ralstonia eutropha H16 by engineering β-oxidation and carbon storage
Source: PeerJ. 2015 Dec 7;3:e1468. doi: 10.7717/peerj.1468 (PMC4675107; doi:10.7717/peerj.1468)

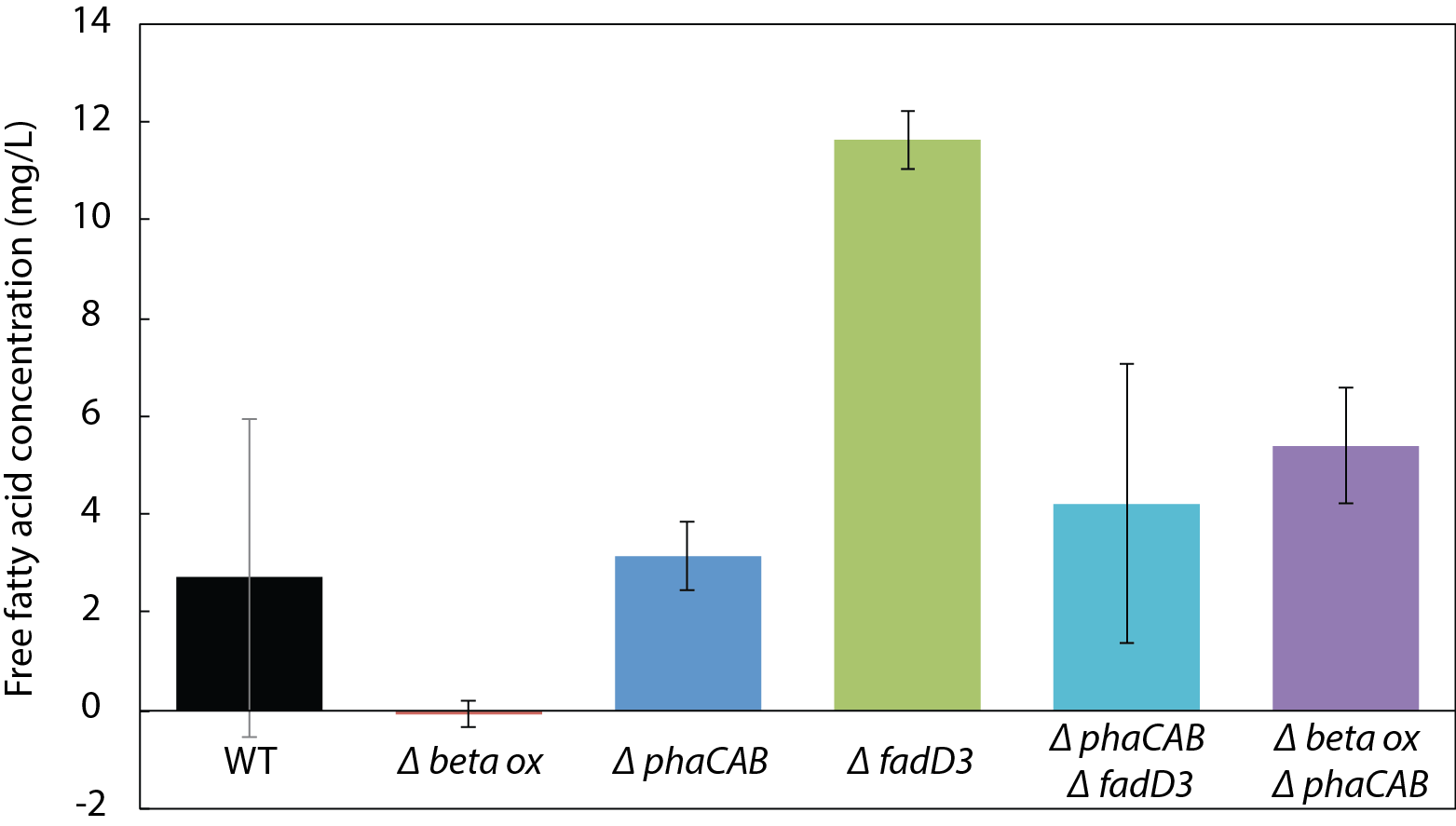

Supplement: Figure S1 — Total free fatty acids (FFA) produced by R. eutropha mutants expressing UcFatB2 in rich broth supernatant 24 h after arabinose induction. Error bars represent the standard error of the mean (S.E.M.) from N ≥ 3 independent experiments. [file peerj-03-1468-s004.png]

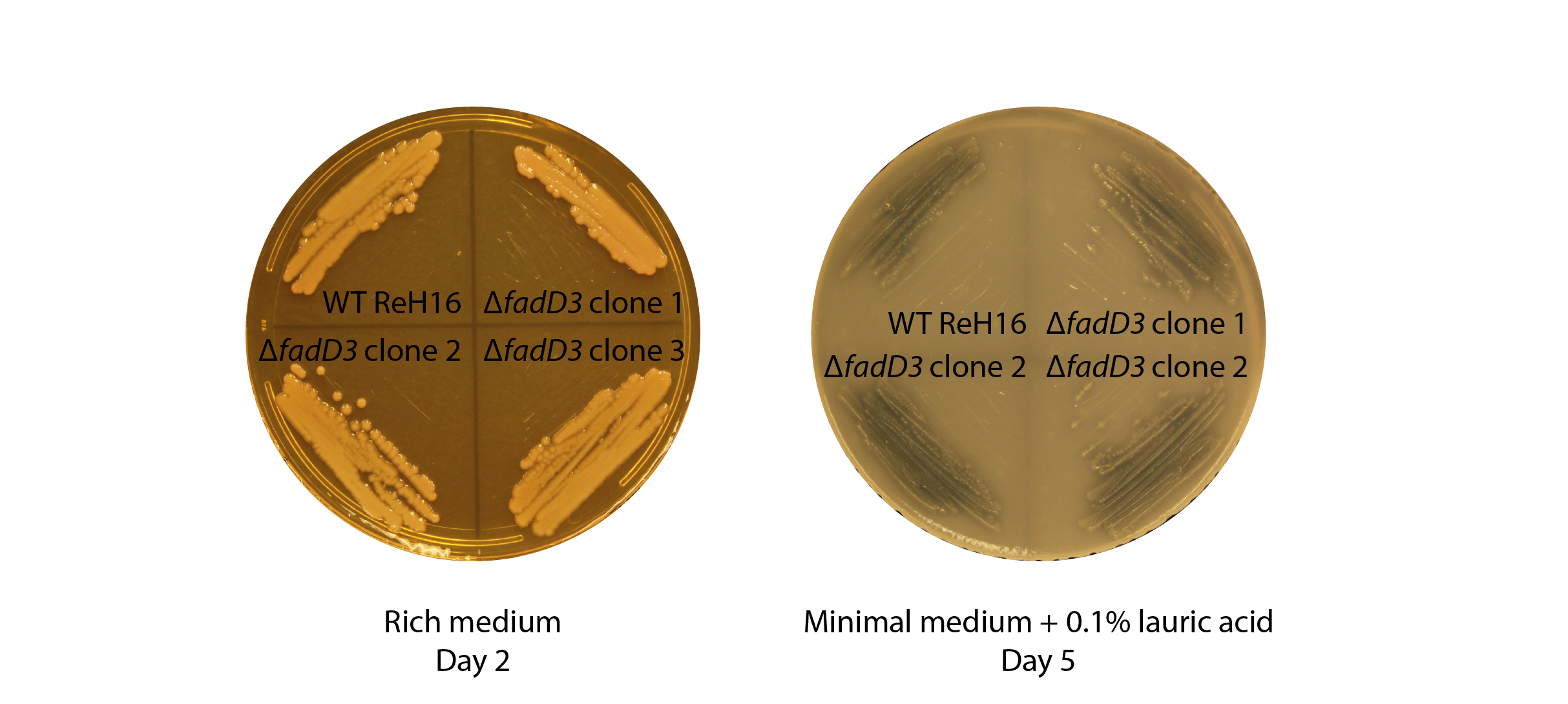

Supplement: Figure S2 — Growth of WT ReH16 and three independent ΔadD3 clones on a (A) rich medium plate after 2 days or (B) minimal medium ±0.1% lauric acid plate after 5 days at 30 °C. [file peerj-03-1468-s006.png]
