# Supplementary material for: Production of fatty acids in Ralstonia eutropha H16 by engineering β-oxidation and carbon storage
Source: PeerJ. 2015 Dec 7;3:e1468. doi: 10.7717/peerj.1468 (PMC4675107; doi:10.7717/peerj.1468)
Supplement: Table S1 [file peerj-03-1468-s007.docx]

**Supplementary Table 1. Strains and Plasmids List**

| **Strain or Plasmid** | **Description** | **Reference** | |
| --- | --- | --- | --- |
| *E. coli* | | |  |
| S17-λ *pir* | Conjugation strain for DNA transfer into *R. eutropha* | Simon *et al.* (35) | |
| *R. eutropha H16* | | |  |
| ReH16 | Wild-type, naturally Gm10 resistant | ATCC17699 | |
| Re2303 | H16Δ(*A0459-A0464*, *A1526-A1531*), Gm10 | Brigham *et al.* (23) | |
| Re2061 | H16Δ*phaCAB*, Gm10 | Lu *et al.* (6) | |
| Re2312 | H16Δ*fadD3*, Gm10 | Brigham *et al.* (23) | |
| JC320 | H16Δ*phaCAB*Δ*fadD3*, Gm10 | This study | |
| JC349 | H16Δ(*A0459-A0464*, *A1526-A1531*)Δ*phaCAB,* Gm10 | This study | |
| JC357 | H16Δ*A0285*, Gm10 | This study | |
| JC358 | H16Δ*A0285*Δ*fadD3*, Gm10 | This study | |
| JT338 | H16Δ*A2794*, Gm10 | This study | |
| JT339 | H16Δ*A2794*Δ*fadD3*, Gm10 | This study | |
| *Plasmids* | | |  |
| pBBR1MCS2 | Broad-Host-Range Cloning Vector, Kan200 | Kovach *et al.* (36) | |
| pRSF-Duet1 | Arabinose-inducible Cloning Vector |  | |
| pCB96 | *fadD3* markerless deletion plasmid | Brigham *et al.* (23) | |
| pJL33 | *phaCAB* markerless deletion plasmid | Lu *et al.* (6) | |
| pJC007 | Arabinose-Inducible Broad-Host-Range Vector pRSF-Ara(Rev)-mCherry | This study | |
| pJC010 | Arabinose-Inducible Broad-Host-Range Vector pBBAD-mCherry | This study | |
| pJC022 | Arabinose-inducible Broad-Host-Range Vector pRSF-Ara(Rev)-C12 thioesterase_RBS14K | This study | |
| pJC023 | Inducible Broad-Host-Range Vector pRSF-Ara(Rev)-C12 thioesterase_RBS42K | This study | |
| pJC024 | Inducible Broad-Host-Range Vector pRSF-Ara(Rev)-C12 thioesterase_RBS66K | This study | |
| pJC025 | Inducible Broad-Host-Range Vector pRSF-Ara(Rev)-C12 thioesterase_RBS84K | This study | |
| pJC043 | *A0285* markerless deletion plasmid | This study | |
| pJC045 | *A2794* markerless deletion plasmid | This study | |
| pJC046 | *B1148* markerless deletion plasmid | This study | |
