# Supplementary material for: Production of fatty acids in Ralstonia eutropha H16 by engineering β-oxidation and carbon storage
Source: PeerJ. 2015 Dec 7;3:e1468. doi: 10.7717/peerj.1468 (PMC4675107; doi:10.7717/peerj.1468)
Supplement: Table S2 [file peerj-03-1468-s008.docx]

**Supplementary Table 2. Primer List**

| **Primer** | **Description** | **Sequence** |
| --- | --- | --- |
| JC014 | *fadD3* diagnostic F primer | TCCTCTACACCTGGCATGAACC |
| JC015 | *fadD3* diagnostic R primer | GCTTTGCCTTCGACCTGAGC |
| JC016 | RBS_14K driving *UcFatB2* F primer | **TCAGTCTAGATGCCAAAAATTAGCACGACCAGGCCAACTACA**ATGACGAATCTCGAATGGAAAC |
| JC017 | RBS_42K driving *UcFatB2* F primer | **TCAGTCTAGAATCCCTAAGAATACTAAAAGGCACTTACA**ATGACGAATCTCGAATGGAAAC |
| JC018 | RBS_66K driving *UcFatB2* F primer | **TCAGTCTAGACACTAGAAAACAAGGAAAGCAACA**ATGACGAATCTCGAATGGAAAC |
| JC019 | RBS_84K driving *UcFatB2* F primer | **TCAGTCTAGATACCTCGCCAAATACTCAGACAGGAGAATATACAA**ATGACGAATCTCGAATGGAAAC |
| JC014 | *fadD3* diagnostic F primer | TCCTCTACACCTGGCATGAACC |
| JC015 | *fadD3* diagnostic R primer | GCTTTGCCTTCGACCTGAGC |
| JC023 | His-tagged *UcFatB2* R primer | /5Phos/CTGTTCGACTTAAGCTAGTGATG |
| JC024 | *phaCAB* diagnostic F primer | ATACATCAGGAAGGTGGCAAC |
| JC025 | *phaCAB* diagnostic R primer | GTCGACGACCTTGAATTCTTCTG |
| JC063 | *A2794* diagnostic F primer | AATGGCTTCGTCGAACTTC |
| JC065 | *A2794* diagnostic R primer | CTGATGGGATTGCTGATG |
| JC066 | *A0285* diagnostic F primer | TACTCGTGCACGAACATG |
| JC067 | *A0285* diagnostic R primer | CACACATCGAACGAGATATC |
| JC068 | *B1148* diagnostic F primer | AATCGGGTCTGCAGGTTC |
| JC069 | *B1148* diagnostic R primer | CATGACCAGAACGTGACATAC |
| JC078 | *B1148* upstream deletion fragment F primer | **CTGATCTAGA**TCCCTGTAAGAATCGTGTAAG |
| JC079 | *B1148* upstream deletion fragment R primer | **GCCGTTAATTAAGCCG**TGCTTGTGTCTCCGCGTATCTG |
| JC080 | *B1148* downstream deletion fragment F primer | **CGGCTTAATTAACGGC**TGGGCTGAAGCGGCGCT |
| JC081 | *B1148* downstream deletion fragment R primer | **TCAGGAGCTC**CATGACCAGAACGTGACATAC |

***bold** = external sequences that do not anneal to the *Ralstonia* genome
